# Depression Literacy, Associated Factors, and Correlation of Related Variables in Middle-Aged Korean Adults: A Cross-Sectional Study

**DOI:** 10.3390/ijerph20116021

**Published:** 2023-05-31

**Authors:** Young Joo Lee

**Affiliations:** The Research Institute of Nursing Science, College of Nursing, Daegu Catholic University, Daegu 42472, Republic of Korea; yjlee39@cu.ac.kr

**Keywords:** adult, depression, health behaviors, health literacy, middle-aged

## Abstract

For the timely treatment and management of depression, a high level of depression literacy (DL) is needed. This study aimed to examine the level of DL and factors associated with DL among middle-aged Korean adults and to verify the relationship between DL, depression, and quality of life (QoL). This cross-sectional study included 485 participants aged 40 to 64 years recruited from five provinces in Korea. DL was measured using a 22-item questionnaire and analyzed by multiple linear regression and correlation analysis. The DL level of the participants was moderate and the correct answer rate was 58.6%. In particular, non-pharmacological treatments, differential symptoms, and pharmacological treatments were low. Out of the participants, 25.2% had depression, and the difference in DL between those with and without depression was not statistically significant. The positive factors associated with DL were being female, having higher education, and being employed. DL was not correlated with depression or psychological QoL. However, higher DL was associated with less heavy drinking, normal body mass index, and not smoking. Improving DL can help individuals seek timely professional help and reduce mental health disparities. Further studies should continue to investigate and develop the association between DL and health-related behaviors as well as depression and QoL to effectively treat and manage depression.

## 1. Introduction

### Background

According to the World Health Organization (WHO), depression is a common mental disorder and it is estimated that 5% of adults worldwide experience depression [1]. Depression can be a serious health problem that may impair proper functioning at home, school, or work. People with recurrent or severe depression can suffer great distress; in the worst cases, it can lead to suicide attempts [1,2,3]. Due to aging, disease, or job loss, men aged 45 to 54 in particular experience stress and depression. Their suicidal ideation is also more than twice compared to that of young adults aged 25 to 34 [4]. Postmenopausal women are more likely than premenopausal women to experience depression [5]. According to a study that used Korean panel data [6], the higher a middle-aged adult’s nutritional, housing, occupational, economic, or healthcare deprivation, the higher their risk of chronic mild depression or severe depression. In Korea [7], the number of patients with depression in 2021 increased by 35.1% compared with that in 2017, with patients in their 40s and 50s making up 27.9% of all patients, and the cost of treatment per person increased by 28.5%. Middle-aged adults encounter crises as a result of various occurrences, including aging, illness, divorce, and retirement. They go through a challenging life transition during midlife and take over crucial societal production tasks at the same time. Therefore, middle-aged depression is not only a burden on individuals, but also on families, friends, coworkers, and nations. To prevent and manage depression properly, everyone needs to enhance depression literacy (DL), a specific type of mental health literacy (MHL).

Firstly, MHL is described as “knowledge and beliefs toward mental health that facilitate recognition, management, and prevention of mental disorders” [8], which reflects an individual’s understanding of the signs and symptoms of mental disorders and the need for professional help. Lee, et al. [9] found that MHL was significantly lower in males than in females. In men, receiving more social support, experiencing higher levels of depression, and being married predicted higher levels of MHL, whereas in women, older age was associated with lower MHL levels. MHL was directly associated with accessing mental health services (e.g., professional help-seeking or counselling) [10,11] by improving help-seeking attitudes [9,12] and behaviors [13]. MHL was positively associated with education, social support, social network, and health status [14]. In particular, MHL was positively correlated with quality of life (QoL) [15]. MHL negatively affected stigma and perceived barriers to care [12]. Therefore, many researchers insist that increasing MHL in communities should be a focus of national policy and population monitoring so that communities as a whole can take measures toward better mental health [9,10,16].

DL, a specific component of MHL, is defined as the ability to recognize symptoms of depression and beliefs about depression, which aids in its management and prevention [17]. Research on DL has been conducted in several countries, including Australia [11], Canada [17], the United States (US) [18], and Iran [2]. The level of DL was reported to be low in these studies. In previous DL studies, participants included immigrants [19,20], older adults [21], hospital staff [2], and students [22]. However, studies on the general population are limited. In particular, the relationship between DL and depression has been inconsistent in previous studies. Bernstein, et al. [20] found that DL and depression were negatively correlated, and lower DL was significantly associated with higher depression. In contrast, Baird, et al. [21] reported that higher DL was significantly associated with higher depression. Moreover, the relationship between DL and depression has not yet been examined in Korea. Therefore, to effectively manage depression and improve mental health, this study aimed to identify the level of DL, the factors associated with DL, and the relationship between DL and related variables among middle-aged adults in Korea.

## 2. Methods

### 2.1. Study Design and Sample

This cross-sectional study investigated DL in community-based adults aged between 40 and 64 years using convenience sampling in five South Korean provinces. The sample size was calculated using the G^*^ Power software version 3.1.9 (Heinrich-Heine-Universität Düsseldorf, Düsseldorf, Germany) for correlation analysis. We chose a small effect size of 0.15 because there were few comparable prior results in the literature [23]. A significance level of 0.05, a power of 0.90, and a two-tailed test were also chosen. As a result, the minimum sample size was determined to be 459 individuals. Assuming a 10% dropout rate, 505 participants were required.

Participants were recruited through a recruitment announcement on the bulletin boards of religious facilities, clubs, and community centers. Participants were enrolled 14–25 July 2022. The notice contained a QR code and website address for accessing the online survey and consent form. The exclusion criteria were taking psychiatric medications and undergoing counseling or cognitive behavioral therapy (CBT) for mental health problems. This is because the participants in this study were healthy people who had no mental illness. A total of 507 questionnaires were collected and 485 surveys (143 males and 342 females) were used for the analysis, excluding 22 surveys with incomplete responses.

### 2.2. Measurements

#### 2.2.1. Depression Literacy (DL)

After obtaining permission for use, DL was measured using the Depression Literacy Questionnaire (D-Lit) developed by Griffiths, et al. [24]. We obtained the Korean translation of the D-Lit from Bernstein, et al. [20] which was used by Korean-Americans. We asked a psychiatric nursing professor and a bilingual (Korean-English) professor to compare the Korean-translated D-Lit with the original D-Lit and consulted them about any unclear Korean translations. Subsequently, the research team reviewed each item and completed the Korean version of the D-Lit. The D-Lit consists of 22 items divided into five domains: incidence and prognosis (three items), differential symptoms (five items), symptom recognition (six items), non-pharmacological effectiveness (four items), and pharmacological knowledge (four items) [19]. Each item was answered using one of the three options: “true”, “false”, or “don’t know.” Each correct “true” or “false” response received one point, and “don’t know” was counted as an incorrect response. The overall score ranged from 0 to 22, with higher scores representing higher DL levels. The internal consistency and test-retest reliability of the original D-Lit were reported to be 0.70 and 0.71, respectively [24]. Cronbach’s α for this study was 0.78.

#### 2.2.2. Depression

Depression was measured using the Korean version of the Patient Health Questionnaire-9 (PHQ-9) translated by Han, et al. [25]. The PHQ-9 was developed by Spitzer, et al. [26]. It consists of nine items answered on a 4-point scale (0 = not at all; 1 = several days; 2 = more than half the days; 3 = nearly every day). Accordingly, the total score ranged from 0 to 27. Higher scores indicated more depressive symptoms. A score of 5 or higher indicated the presence of depression [25]. In this study, Cronbach’s α for the PHQ-9 was 0.84.

#### 2.2.3. Psychological QoL

QoL was measured using the Korean version of the World Health Organization Quality of Life Scale-Abbreviated version (WHOQOL-BREF), translated by Min, et al. [27]. The WHOQOL-BREF was developed by the World Health Organization [28]. This measure consists of 24 items in four domains (physical health, psychological health, social relationships, and environment); additionally, two items measure the overall perception of health. In this study, only six items from the psychological domain were used. The psychological QoL score was calculated by multiplying the mean score of the six items by 4 [28]. Total scores ranged from 4 to 20, with higher scores indicating a higher psychological QoL. In this study, Cronbach’s α for this scale was 0.85.

#### 2.2.4. Health-Related Behaviors

We collected data on current smoking status, as well as height and weight from which body mass index (BMI) could be calculated. We asked the participants whether they exercised enough to sweat two to three times per week. They were also asked whether they had any chronic diseases such as hypertension, diabetes mellitus (DM), cardio-cerebrovascular diseases, or malignant tumors in the past 12 months. Heavy drinking was evaluated using the Korean version of the Alcohol Use Disorders Identification Test (AUDIT-K) which was developed and verified by Lee, et al. [29]. The cut-off point for heavy drinking was defined as a score of ≥10 for men and ≥6 for women.

### 2.3. Data Analysis

The association between general characteristics and DL was analyzed using an independent *t*-test or analysis of variance (ANOVA). Multiple linear regression analysis was performed to analyze factors associated with DL. Correlations between DL and related variables were determined using the Pearson or Spearman correlation coefficient. Statistical significance was set at *p* < 0.05. All statistical tests were performed using SPSS version 25.0 software (IBM, Armonk, NY, USA).

## 3. Results

### 3.1. DL Level

The percentages of correct answers for each item of the D-Lit are presented in Table 1. The average percentage of correct answers was 58.6%. The domain with the highest DL level was incidence and prognosis (87.2%), and the item with the highest percentage of correct answers in this domain was “Many famous people have suffered from depression” (91.1%). The domain with the lowest DL level was non-pharmacological effectiveness (30.7%), and the item with the lowest percentage of correct answers in this domain was “Counseling is as effective as CBT for depression” (3.3%). The domain with the second-lowest DL level was differential symptoms (41.9%), and the item with the lowest percentage of correct answers in this domain was “Having several distinct personalities may be a sign of depression” (27.6%). The domain with the third-lowest DL level was pharmacological knowledge (51.6%), and the item with the lowest number of correct answers in this domain was “Antidepressants are addictive” (29.3%).

### 3.2. Participants’ General Characteristics and Differences in DL

The participants’ general characteristics are shown in Table 2. Women accounted for 70.5% of the sample; by age group, 50.5% of the participants were in their 40s, 40.6% were in their 50s, and 8.9% were in their 60s. Approximately 60% of the participants had a college or university degree, and 29.9% had a master’s degree or higher. In terms of employment, 77.1% of the participants had a job, and half lived in the capital areas of Seoul and Gyeonggi. Approximately 50% of the participants had a normal BMI, 19.0% reported heavy drinking, and 54.2% exercised regularly.

Differences in DL according to general characteristics are shown in Table 2. The mean DL score in this study was 12.89 ± 3.94 (min = 0, max = 21). The DL score was higher in women than in men (*t* = −4.56, *p* < 0.001), as well as in those with a higher education level (F = 18.28, *p* < 0.001), were employed (*t* = −2.18, *p* = 0.030), or lived in the capital area (F = 3.37, *p* = 0.018). In addition, the DL score was lower in participants with DM (*t* = 2.37, *p* = 0.018), current smokers (*t* = 2.12, *p* = 0.035), and heavy drinkers (t = 1.98, *p* = 0.048). However, the difference in DL between participants with depression and those without depression was not statistically significant.

### 3.3. Factors Associated with DL

The factors associated with DL are presented in Table 3. DL increased significantly in women compared with men (Β = 2.10, *p* < 0.001), and among participants with college or university education (Β = 2.19, *p* < 0.001), those with a master’s or higher degree (Β = 3.32, *p* < 0.001), and those with a job (Β = 1.32, *p* = 0.004). The factor determination value was 12.0%.

### 3.4. Correlation between DL and Related Variables

The correlations between DL and related variables are presented in Table 4. DL was not correlated with depression or psychological QoL. Depression was negatively correlated with psychological QoL (r = −0.548, *p* < 0.001). However, a higher DL was associated with less heavy drinking (r = −0.112, *p* = 0.013), normal BMI (r = 0.093, *p* = 0.040), and not currently smoking (r = −0.114, *p* = 0.012). Higher depression was associated with higher levels of heavy drinking (r = 0.185, *p* < 0.001), chronic disease (r = 0.096, *p* = 0.035), current smoking (r = 0.101, *p* = 0.026), and not engaging in regular exercise (r = −0.193, *p* < 0.001). Higher psychological QoL was associated with less heavy drinking (r = −0.090, *p* = 0.048), normal BMI (r = 0.187, *p* < 0.001), and engagement in regular exercise (r = 0.274, *p* < 0.001).

## 4. Discussion

### 4.1. The Level of DL

In this study, we obtained an average DL score of 12.89. This result was higher than the average score of 9.4 among Korean Americans in the US [20] or the score of 11.62 among hospital staff in Iran [2]. According to the classification of DL in Oh, et al. [19], the correct answer rate in this study was the highest for incidence and prognosis of depression, and the lowest for non-pharmacological effectiveness of depression and differentiating symptoms of depression. This could mean that participants lacked knowledge and understanding of the differentiation and treatment of depression rather than its incidence, which is consistent with previous studies [2,19]. However, we found that the result of 87.2% for the incidence and prognosis domain was approximately twice as high as the 49.7% [2] and 45.1% [19] reported in previous studies. In particular, the top answer rate of 91.1% was for the item “Many famous people have suffered from depression”. According to a study conducted in Korea [30], when the media covered a celebrity suicide that was the result of depression in a particular month, more people visited a psychiatrist for depression in the following month. People learn from news about celebrities that depression can occur in anyone, and they can also obtain information on the prevention and treatment of depression.

In the non-pharmacological effectiveness domain, which had the lowest correct answer rate, a majority of the participants in this study lacked knowledge about CBT. Almost all the participants (96.7%) mistakenly believed that counseling was as effective as CBT for depression, and only 55.5% knew that CBT was as effective as antidepressants for mild-to-moderate depression. These results are consistent with those of a previous study [2]. According to a cohort study conducted on patients with major depressive disorders [31], the CBT group showed significantly decreased depressive symptoms after the intervention compared with the support-counseling-pill placebo group. In a recent network meta-analysis [32], CBT was shown to be effective in treating acute depression. CBT can also be effectively delivered through individual and group therapy, as well as through telephone-administered and guided self-help. This demonstrates the need to disseminate CBT knowledge that has already been validated in the medical field to the general public.

In the differential symptoms domain, which had the second-lowest correct answer rate, 72.4% of participants incorrectly cited a distinct personality as a symptom of depression, and 56.1% believed that reckless and foolhardy behavior was a common sign of depression. According to Wang, et al. [17], men were more likely than women to perceive depression as being caused by character weakness. The inability to differentiate symptoms from other mental health problems can lead to prejudice against patients with depression and delay the patient’s timing of treatment. This indicates the need for male adults’ DL education. In Canada [33], a website for men with depression was designed to leverage male preference for independence, autonomy, and self-sufficiency while conveying a message that encouraged men to seek professional help. It is necessary to develop men-friendly methods for raising awareness about male depression and literacy intervention.

In the pharmacological knowledge domain, which had the third-lowest correct answer rate, most participants had misconceptions about antidepressants. Out of the participants, 70.7% believed that antidepressants were addictive and 58.6% answered that antidepressant medications usually work straight away. The use of antidepressants is less likely among older Korean Americans who associate depression with family shame [34]. In particular, it has been reported that antidepressants are significantly less likely to be used by low-income, uninsured patients, and racial and ethnic minorities [35], so it is necessary to pay attention to providing treatment opportunities for depression in vulnerable populations. However, according to Beshai, et al. [36], there were no perceptual changes regarding antidepressant medicine after psychoeducation. Therefore, mental health practitioners should provide accurate and continuing education to patients and the public regarding the efficacy of, and misconceptions and erroneous beliefs about, antidepressant use for the right treatment of depression.

### 4.2. Factors Associated with DL

We found that the factors associated with DL in this study were sex, education, and job status. This is consistent with the results of previous studies. Women are more affected by depression than men are [1], even focusing on biological factors such as hormonal changes (e.g., menstrual cycle, pregnancy, menopause) [37], but women have higher DL levels than men do [2]. Additionally, people with higher educational levels have high levels of DL [2,20,38]. Ho, et al. [39] reported that finding a job was effective in alleviating or reversing depression symptoms. However, in interviews with unemployed persons, Samkange-Zeeb, et al. [40] identified fear of the side effects of psychopharmacological treatment, perceived discrimination by mental healthcare professionals, and general practitioners’ lack of interest as barriers to MHL. Mental health practitioners need to play an important role as sources of health information and thoughtful advice for the unemployed.

Strengthening DL is an important strategy to alleviate social exclusion and reduce health inequality regarding depression in vulnerable populations, such as those with low education or the unemployed. DL can be improved through DL interventions. In a study among adolescents in Malaysia [41], there was a significant improvement in variables (better depression literacy, reduced self-stigma, and increased help-seeking attitude) in the intervention group compared with the control group at the post-test and three-month follow-ups. Additionally, in a study of 54 high school students in the US [22], there was a significant effect of the Adolescent Depression Awareness Program on DL, with intervention students achieving DL at higher rates than controls four months later.

### 4.3. Relationship between DL and Related Variables

In this study, there was no correlation between DL and depression. Previous studies have shown inconsistent results; lower DL was correlated with higher depression [20], or higher DL was significantly associated with higher depression [21]. A low DL makes it difficult to recognize depression quickly and accurately. However, one should not interpret this to mean that people with high DL never have high levels of depression because of the early detection or maintenance of low depression with appropriate treatment. DL is an understanding of depression, which is an emotion that requires management and treatment. To date, the causal relationship remains unclear. However, it is important to note that greater knowledge is significantly associated with more positive attitudes toward mental illness and professional help-seeking behaviors [39]. In a study of Korean American parents of adolescent children [18], DL showed a positive correlation with attitudes toward service use and had partial mediating and moderating effects on the relationship between personal stigma and attitudes toward service use. Thus, everyone needs to have a high DL to prevent, treat, and manage depression at the appropriate time. Therefore, when someone is depressed, they or someone close to them will recognize it and help or treatment can be obtained [21]. Additional research is required to verify the relationship between DL and depression.

There was no statistically significant correlation between DL and psychological QoL. Although studies on the correlation between DL and QoL are scarce, MHL was found to be positively correlated with QoL in Iran’s general population [15]. Specifically, those who received information about their mental illness had high MHL and QoL. Further studies are required to confirm the relationship between DL and QoL.

However, in this study, we found a statistically significant relationship between DL and health-related behaviors. Higher DL was associated with less heavy drinking, normal BMI, and not smoking. This could mean that people with high DL are better at health-related behaviors, such as not smoking, reducing alcohol consumption, and weight management. Although there are reports that self-reported BMI is not a useful predictor of health due to under-reporting [42], BMI is a simple calculation to evaluate the current health status of a large population. According to Wang, et al. [17], men were more likely than women to use alcohol to cope with depression. Walters, et al. [43] reported that higher health literacy leads to changes in health behaviors such as avoidance of drugs, alcohol, and opiates, increased physical activity, smoking cessation, and cancer screening. Improving DL is believed to make lifestyles more manageable. Consequently, improved health behaviors may result in improved depressive symptoms. Future research should continue to verify the relationship between DL and health-related behaviors.

### 4.4. Study Limitations

This study has several limitations. Convenience sampling may have led to selection bias. Because the study sample was mainly gathered from religious facilities, clubs, and communities, it is possible that participants with relatively good social activities and social support were recruited. In addition, the majority of the participant demographics were female, highly educated, and urban, so it is necessary to attempt studies in a homogenous sample or in a different setting. Therefore, the findings cannot be generalized to other middle-aged Korean adults. Additionally, because this study was conducted online, only participants familiar with mobile technology use were included. Finally, we did not exclude healthcare providers from being participants; therefore, participants may have included healthcare providers with good psychiatric experience and knowledge within a general population, such as doctors, nurses, clinical psychology counselors, or social workers.

## 5. Conclusions

This study is the first to examine middle-aged Koreans’ perspectives on depression literacy. The findings emphasize the need to improve DL. The participants’ DL level was moderate, and we found that the factors associated with DL were being female, having higher education, or having a job. Understanding the level of DL and the factors associated with it can provide mental health practitioners with the insights they need to prevent and manage depression in their communities. Although DL has not been found to be correlated with depression or QoL, we identified a correlation between DL and health-related behaviors. Future research should continue to investigate and develop the relationships between DL and other variables to prevent and manage depression.

## Figures and Tables

**Table 1:** Distribution of correct answer rates for each item on the depression literacy questionnaire.

Items (Correct Answer)	% of Correct Answer
**Incidence and prognosis (3 items)**	**87.2**
Many famous people have suffered from depression. (True)	91.1
Moderate depression disrupts a person’s life as much as multiple sclerosis or deafness. (True)	86.0
Most people with depression need to be hospitalized. (False)	84.5
**Differential symptoms (5 items)**	**41.9**
Not stepping on cracks in a footpath may be a sign of depression. (False)	59.2
People with depression often hear voices that are not there. (False)	45.2
Reckless and foolhardy behavior is a common sign of depression. (False)	43.9
People with depression often speak in a rambling and disjointed way. (False)	33.6
Having several distinct personalities may be a sign of depression. (False)	27.6
**Symptom recognition (6 items)**	**81.4**
Loss of confidence and poor self-esteem may be a symptom of depression. (True)	89.7
People may move more slowly or become agitated as a result of their depression. (True)	86.6
People with depression may feel guilty when they are not at fault. (True)	83.9
Depression does not affect your memory and concentration. (False)	83.1
Eating too much or losing interest in food may be a sign of depression. (True)	76.5
Sleeping too much or too little may be a sign of depression. (True)	68.9
**Non-pharmacological effectiveness (4 items)**	**30.7**
Cognitive behavioral therapy is as effective as antidepressants for mild to moderate depression. (True)	55.5
Of all the alternative and lifestyle treatments for depression, vitamins are likely to be the most helpful. (False)	38.8
Many treatments for depression are more effective than antidepressants. (False)	25.4
Counseling is as effective as cognitive behavioral therapy for depression. (False)	3.3
**Pharmacological knowledge (4 items)**	**51.6**
People with depression should stop taking antidepressants as soon as they feel better. (False)	74.8
Clinical psychologists can prescribe antidepressants. (False)	61.0
Antidepressant medications usually work straight away. (False)	41.4
Antidepressants are addictive. (False)	29.3
**Total**	**58.6**

**Table 2:** Differences in depression literacy according to general characteristics (n = 485).

Variables		Categories	N (%) or M ± SD (Min-Max)	Depression Literacy	
				M ± SD (Min-Max)	t/F (*p*), Sheffé *
Demographic factors	Sex	Male Female	143 (29.5) 342 (70.5)	11.68 ± 3.84 13.41 ± 3.87	−4.56 (<0.001)
	Age (years)	40–49	50.05 ± 6.25 (40.0–64.0) 245 (50.5)	12.89 ± 4.13	0.00 (1.000)
		50–59	197 (40.6)	12.89 ± 3.87	
		60–64	43 (8.9)	12.91 ± 3.12	
	Marital status	No spouse	61 (12.6)	13.26 ± 4.24	0.78 (0.434)
		Having a spouse	424 (87.4)	12.84 ± 3.90	
	Education	≤High School ^a^	50 (10.3)	10.30 ± 4.01	18.28 (<0.001), a < b < c
		College/University ^b^ ≥Master’s degree ^c^	290 (59.8) 145 (29.9)	12.77 ± 3.68 14.03 ± 3.99	
	Income (million KRW)	<3.0	132 (27.2)	12.42 ± 3.99	1.35 (0.261)
		3.0–6.9	242 (49.9)	13.04 ± 4.01	
		≥7.0	111 (22.9)	13.14 ± 3.69	
	Job	No Yes	111 (22.9) 374 (77.1)	12.18 ± 3.65 13.10 ± 4.00	−2.18 (0.030)
	Residence	Seoul/Gyeonggi ^a^ Chungcheong/Gangwon ^b^ Gyeongsang ^c^	232 (47.8) 61 (12.6) 117 (24.1)	13.29 ± 4.01 11.66 ± 3.81 13.08 ± 3.68	3.37 (0.018), a > b
		Jeolla ^d^	75 (15.5)	12.37 ± 4.02	
Health-related factors	BMI (kg/m^2^)	Underweight (<18.5)	20 (4.1)	12.80 ± 4.72	1.26 (0.287)
		Normal (18.5–22.9) Overweight (23.0–24.9)	245 (50.5) 108 (22.3)	13.20 ± 3.96 12.34 ± 3.82	
		Obese (≥25.0)	112 (23.1)	12.76 ± 3.84	
	Hypertension	No Yes	407 (83.9) 78 (16.1)	12.94 ± 3.99 12.65 ± 3.67	0.58 (0.559)
	DM	No Yes	460 (94.8) 25 (5.2)	12.99 ± 3.92 11.08 ± 3.98	2.37 (0.018)
	Cardio-cerebrovascular disease	No Yes	473 (97.5) 12 (2.5)	12.93 ± 3.92 11.42 ± 4.74	1.32 (0.189)
	Malignant tumor	No Yes	474 (97.7) 11 (2.3)	12.86 ± 3.89 14.09 ± 5.74	−1.02 (0.308)
	Current smoking	No Yes	443 (91.3) 42 (8.7)	13.01 ± 3.96 11.67 ± 3.50	2.12 (0.035)
	Heavy drinking	No Yes	393 (81.0) 92 (19.0)	13.06 ± 3.98 12.16 ± 3.71	1.98 (0.048)
	Regular exercise	No Yes	222 (45.8) 263 (54.2)	13.06 ± 3.74 12.75 ± 4.10	0.87 (0.382)
Depression		No Yes	363 (74.8) 122 (25.2)	12.77 ± 4.04 13.25 ± 3.61	−1.15 (0.253)
Total				12.89 ± 3.94 (0.0–21.0)	

M = Mean; SD = Standard Deviation; KRW = South Korean Won; BMI = Body mass index; DM = Diabetes mellitus; * Post hoc (Scheffé) after ANOVA test represents the significant difference for each group ^a^, ^b^, ^c^ and ^d^.

**Table 3:** Factors associated with depression literacy.

Variables		Β	SE	*β*	*t*	*p*
Constant		7.80	1.10		7.10	<0.001
Sex	Female ^a^	2.10	0.45	0.24	4.63	<0.001
Age (years)	40~49	−0.61	0.65	−0.08	−0.94	0.347
	50~59	−0.15	0.65	−0.02	−0.23	0.821
	60~64	reference				
Having a spouse	Yes ^b^	−0.14	0.53	−0.01	−0.27	0.789
Education	≤High school	reference				
	College/University	2.19	0.58	0.27	3.78	<0.000
	≥Master’s degree	3.32	0.64	0.39	5.17	<0.000
Income ^c^	<3.0	reference				
	3.0–6.9	0.83	0.43	0.11	1.91	0.057
	≥7.0	0.70	0.53	0.07	1.33	0.184
Job	Yes ^b^	1.32	0.45	0.14	2.93	0.004
Residence	Capital ^d^	0.29	0.36	0.04	0.81	0.418
BMI	Normal ^e^	0.38	0.35	0.05	1.07	0.285
Chronic disease ^f^	Yes	0.26	0.42	0.03	0.63	0.527
Current smoking	Yes ^b^	0.25	0.68	0.02	0.36	0.715
Heavy drinking ^g^	Yes	−0.65	0.45	−0.07	−1.45	0.148
Regular exercise	Yes ^b^	−0.16	0.34	−0.02	−0.45	0.650
Adj R^2^ = 0.12, *p* < 0.001						

^a^ reference = Male. ^b^ reference = No. ^c^ unit = million South Korean Won. ^d^ Capital = Seoul/Gyeonggi; Non-capital = Chungcheong/Gangwon, Gyeongsang, and Jeolla; reference = Noncapital. ^e^ BMI = body mass index; abnormal = underweight, overweight, and obese; reference = abnormal. ^f^ At least one of four diseases (hypertension, diabetes mellitus, cardio-cerebrovascular disease, or malignant tumor); reference = No. ^g^ AUDIT-K score is ≥10 for men and ≥6 for women; reference = no.

**Table 4:** Correlation between depression literacy and related variables.

Variables	Depression Literacy	Depression	Psychological QoL
Depression literacy	-	-	-
2. Depression	0.043 (0.347) ^a^	-	-
3. Psychological QoL	−0.029 (0.517) ^a^	−0.548 (<0.001) ^a^	-
4. Heavy drinking	−0.112 (0.013) ^a^	0.185 (<0.001) ^a^	−0.090 (0.048) ^a^
5. BMI	0.093 (0.040) ^b^	−0.070 (0.124) ^b^	0.187 (<0.001) ^b^
6. Chronic disease	−0.016 (0.720) ^b^	0.096 (0.035) ^b^	−0.065 (0.151) ^b^
7. Current smoking	−0.114 (0.012) ^b^	0.101 (0.026) ^b^	−0.050 (0.271) ^b^
8. Regular exercise	−0.034 (0.457) ^b^	−0.193 (<0.001) ^b^	0.274 (<0.001) ^b^

1. Depression literacy questionnaire score. 2. Patient Health Questionnaire-9 (PHQ-9) score. 3. QoL = Quality of Life, WHOQOL-BREF score. 4. Korean version of Alcohol Use Disorders Identification Test (AUDIT-K) score. 5. BMI = Body mass index; normal = 1; abnormal (underweight, overweight, or obese) = 0. ^a^ Pearson correlation coefficient. ^b^ Spearman correlation coefficient, Yes = 1, No = 0.

## Data Availability

The datasets analyzed in this study are available from the corresponding author on reasonable request.
